# A cell-free strategy for host-specific profiling of intracellular antibiotic sensitivity and resistance

**DOI:** 10.1038/s44259-023-00018-z

**Published:** 2023-12-18

**Authors:** Kameshwari Chengan, Charlotte Hind, Maria Stanley, Matthew E. Wand, Lakshmeesha K. Nagappa, Kevin Howland, Tanith Hanson, Rubén Martín-Escolano, Anastasios D. Tsaousis, José A. Bengoechea, J. Mark Sutton, Christopher M. Smales, Simon J. Moore

**Affiliations:** 1 School of Biosciences, Division of Natural Sciences, University of Kent, Canterbury, CT7 2NJ United Kingdom; 2 School of Biological and Behavioural Sciences, Queen Mary University of London, London, E1 4NS United Kingdom; 3 Technology Development Group, Research and Evaluation, UK Health Security Agency, Salisbury, SP4 0JG United Kingdom; 4 Wellcome-Wolfson Institute for Experimental Medicine, Queen’s University Belfast, Belfast, BT9 7BL United Kingdom; 5 National Institute for Bioprocessing Research and Training, Blackrock Co, Dublin, Ireland

**Keywords:** Bacterial techniques and applications, Ribosome, Enzyme mechanisms

## Abstract

Antimicrobial resistance (AMR) is a pandemic spread across multiple infectious disease-causing microbes. To provide a host-specific tool to study antibiotic susceptibility and resistance, here we develop *Klebsiella pneumoniae* cell-free gene expression (CFE) systems from laboratory and clinical isolates. Using proteomics, we identify relative differences and unique proteins for these new CFE systems in comparison to an *Escherichia coli* MG1655 CFE model. Then we profile antimicrobial susceptibility in parallel with whole cells to quantify CFE antibiotic potency. Finally, we apply this native CFE tool to study AMR variants at a proof-of-concept level. Definably we show that RpoB H526L confers a 58-fold increase in CFE resistance to rifampicin—a genotype observed in rifampicin-resistant *Mycobacterium tuberculosis* clinical isolates. Overall, we provide a cell-free synthetic biology strategy for the profiling of antibiotic sensitivity and resistance from *K. pneumoniae*. While initial extract processing requires Biosafety Level 2, the CFE system is non-living, suitable for long-term storage and study in a Biosafety Level 1 lab. We anticipate the *K. pneumoniae* CFE bioassay is advantageous for host-specific antimicrobial testing, the characterisation of intracellular AMR variants and potentially structure-activity relationship studies.

## Introduction

*Klebsiella pneumoniae* (and *K. oxytoca*) is a key cause of hospital-acquired bacteriaemia deaths, with carbapenem-resistant strains carrying a mortality rate of up to 41%^1^. Distinctly, *K. pneumoniae* presents a “phalanx” of cell envelope defences that act as a barrier against many antibiotic classes^2^. This includes an extracellular capsular polysaccharide layer^3^ that also acts as a virulence factor^4–6^. Like other Gram-negative bacterial pathogens, *K. pneumoniae* is linked to rising levels of pathogenicity and is increasingly challenging to treat with frontline antibiotics due to the rise of antimicrobial resistance (AMR). Hence, there is a need to develop new therapeutics, as well as consider how resistance develops to both current and future antibiotics. To study AMR, there are two distinct approaches: genotyping and phenotyping. Genotyping uses whole-genome sequencing (WGS) and bioinformatics tools, such as RESfinder^7,8^, to detect genetic AMR determinants (i.e., resistance genes, single-nucleotide polymorphisms). This analysis is reliant upon laboratory-based phenotypic experiments such as whole-cell growth inhibition and time killing assays. However, these assays are a complex measure of antibiotic import, efflux, metabolism, and both on- and off-target interactions, all overlaid by mutations associated with the resistome^9^. To inspect individual AMR mechanisms and antibiotic activity for structure-activity relationship (SAR) studies, requires the isolation of recombinant proteins or complexes (e.g., ribosomes) through routine structural biology and biochemistry techniques. However, these in vitro approaches are research intense, while as single isolated targets, they lack the associated macromolecular components and interactions that naturally occur within a cell.

Alternatively, cell-free synthetic biology is an emerging research discipline that offers a systems-level biochemical approach to explore the intracellular molecular machinery of microbes^10–13^. Cell-free systems derived from cell extracts have the endogenous RNA and metabolite pool, and up to ~1892 detectable proteins^14^ including components from protein synthesis^15,16^, transcription^17^ and metabolism^18–22^. Conceptually distinct, cell-free offers several advantages over traditional whole-cell experiments, particularly a reduced timescale and increased throughput. While CFE systems are devoid of genomic DNA and a cell envelope, they are non-living and safe to study at Biosafety Level 1 (BSF-1). This allows CFE systems to be frozen for long-term storage or freeze-dried for shipping for direct field testing, as seen with viral diagnostics^23,24^. In considering AMR and antimicrobials, cell-free approaches have been used for mode of action studies to detect ribosomal inhibitors^25,26^. For example, a high-throughput (HT) *Escherichia coli* in vitro translation assay was used to screen ~25,000 natural product extracts to identify a novel tetrapeptide (GE81112) ribosomal inhibitor^27^. However, these studies are limited to non-pathogenic models such as *Escherichia coli* and *Bacillus subtilis*^25,26^, with the exception of a *Streptococcus pneumoniae* CFE system^28,29^.

Recently, we and others have shown the potential of CFE to explore a range of cell types^22,30–35^ as a native biochemical model. In comparison to model *E. coli* CFE systems^10,12,15,36,37^, non-model CFE systems differ in terms of metabolism and genetics^31,34^, providing an advantage to study native genetic elements^31^ and biosynthetic pathways^32^ from non-model microbes. However, the cell-free proteome and metabolism are less characterised. Even for established *E. coli* CFE, the extract varies between strains and processing methods, which influences CFE activity^38^. *E. coli* CFE catabolises a range of glycolysis metabolites (and glutamate) to regenerate ATP, which leverages prolonged protein synthesis^10,18^. Interestingly, a *Priesta megaterium* CFE system was shown to use succinate as a sole energy source^31^, which is not reported in other systems. CFE reactions also have active energy and amino acid metabolic pathways^14,39^ and accumulate inhibitory waste products (e.g., lactate, acetate)^38^. While understudied and unique to each cell type, underlying all these biochemical processes is a myriad of background protein-protein interactions (PPIs), a potential alternative target for antimicrobial development^40,41^.

Here we describe a cell-free synthetic biology strategy to explore an important infectious disease-causing microbe. Specifically, we develop a CFE system from *K. pneumoniae*, a major World Health Organisation (WHO) priority pathogen. We initially use this as a tool to study antibiotic target susceptibility and resistance in this non-living biochemical model. We optimise CFE systems from *K. pneumoniae* ATCC 13882 (laboratory model) and two clinical strains (ST258-T1b and NJST258-1) to show the generalisability of the technique. To characterise the CFE systems, we apply label-free high-resolution mass spectrometry to identify common and unique proteins in comparison to *E. coli* MG1655. We then characterise the *K. pneumoniae* ATCC 13882 CFE and whole cells to reveal key differences in antibiotic activity. Finally, we show the use of this native CFE tool to study intracellular AMR variants at a proof-of-concept level. Specifically, we study three isogenic mutants (derived from the ATCC 13822 strain) with elevated resistance to single antibiotics. Whilst the removal of the cell envelope and genomic DNA is a limitation of CFE, it is also advantageous since it allows us to directly study gene expression antimicrobial targets in isolation within the native cytoplasmic extract of *K. pneumoniae*.

## Results

### Establishing a rapid and safe *K. pneumoniae* CFE platform from laboratory and clinical isolates

**Fig. 1:**
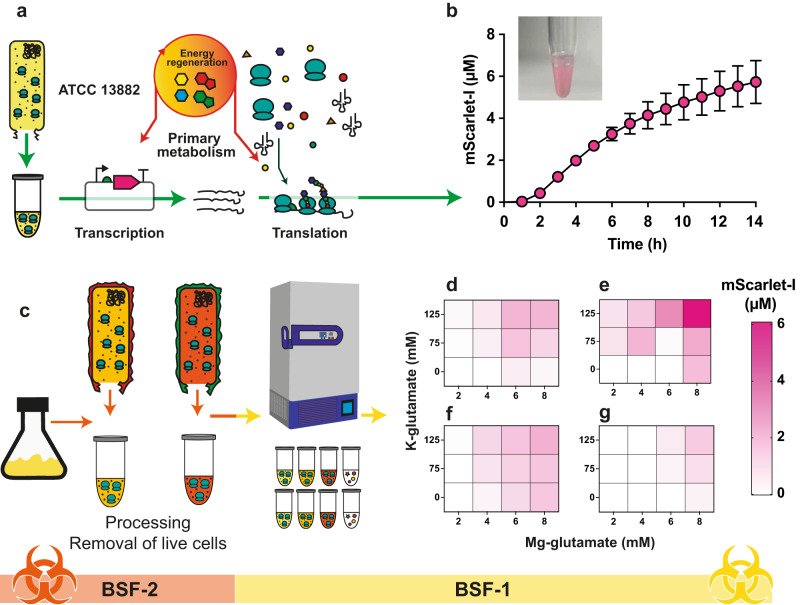
A *K. pneumoniae* CFE platform from laboratory and clinical isolates. **a** Overview of components and stages of the CFE reaction. **b** *K. pneumoniae* ATCC 13882 CFE activity of batch reactions (visual images) and real-time measurement of mScarlet-I (μM) concentration (calculated from a reference standard) over 14 h. Data is the mean and standard deviation of two biological repeats. **c** Workflow for CFE extract processing of clinical strains. **d**, **e** A heatmap representing the optimisation of mScarlet-I (μM) synthesis from ST258-T1b and **f**, **g** NJST258-1 clinical CFE systems with Mg-glutamate and K-glutamate. Two independent repeats (left and right panels) are shown from four biological repeats. Error bars represent the standard deviation of three measurements for each extract.

A range of CFE systems have recently emerged as a fresh approach to study non-model microbes^22,30–32,34,35^. To make active CFE systems, this ideally requires a cell extract obtained from rapidly dividing cells when the ribosome copy number peaks^42,43^. Then, a gene expression reporter system (i.e., plasmid DNA) and a metabolite solution are added to the cell extract to initiate mRNA transcription, translation and metabolism (Fig. 1a). To generate a *K. pneumoniae* CFE system, we selected the ATCC 13882 standard strain, which is used for quality control in antimicrobial susceptibility testing^44^. We then optimised the growth of *K. pneumoniae* ATCC 13882 (Supplementary Figs. 1–3) and processing steps to provide concentrated cell extracts (20–24 mg/mL). Initially, we tested a range of standard plasmids, including Pr-deGFP-MGapt (Supplementary Table 1), which is active in *E. coli*—i.e., closely related to *K. pneumoniae*. However, we were unable to detect protein synthesis. Next, we tested the pTU1-A-SP44-*mScarlet-I* (pSJM1174) plasmid that is optimised for *Streptomyces* gene expression^45^. This plasmid contains a constitutive promoter driving the transcription of the encoded mScarlet-I fluorescence protein. We observed peak activity with cells harvested at the exponential phase (Supplementary Fig. 4) and through optimisation (Supplementary Figs. 5–10), mScarlet-I synthesis reached up to 5.73 ± 1.02 µM (Fig. 1b). Maximum mScarlet-I levels reached 11 µM (Supplementary Fig. 11), although typical yields were around ~6 μM. While protein synthesis rates were modest (~0.5 aa/s) compared to *E. coli* CFE (>1.5 aa/s^10^), the reactions were active for up to 14–16 h. In our experience, new CFE systems are typically only active for ~2–6 h.

*K. pneumoniae* requires handling in Biosafety Level 2 (BSL-2) facilities. Importantly, CFE systems are non-living^10^ and stable when frozen or freeze-dried^23^. To test cell viability, we incubated the cell extracts to assess for potential colony-forming units (CFU). We found approximately 10^2^ CFUs per mL of concentrated cell extract (Supplementary Fig. 12). However, these viable cells were removed by passing the cell extracts (20 mg/mL) through a 0.2 µm filter without altering CFE activity (Supplementary Fig. 13). Removal of viable cells potentially allows the transfer of cell extracts from a CL2 laboratory, for storage at −80 °C. Next, to demonstrate the generalisability of CFE to study other strains, we repeated the process with the multi-antibiotic-resistant ST258-T1b and NJST258-1 clinical isolates (Fig. 1c). We obtained reasonable CFE activity for these clinical strains after optimising the magnesium glutamate and potassium glutamate levels. Magnesium is required for the activity of ATPases and ribosomes. Glutamate provides additional energy through the Krebs cycle via α-ketoglutarate^46^. This metabolite is highly abundant (~96 mM) inside *E. coli* cells and is a key source of energy and nitrogen^47^. In comparison to the ATCC 13882 strain, NJST258-1 and ST258-T1b CFE systems produced less mScarlet at ~2 µM and ~4.5 μM (*n* = 2 biological repeats), respectively (Fig. 1d–g). At this point, we wanted to explore antibiotic resistance in these clinically relevant CFE systems. However, we did encounter issues with antibiotic resistance and variable activity between batches (Supplementary Fig. 14). This may have been due to increased cell buoyancy (in comparison to ATCC 13882) during cell harvesting and extract preparation steps. To compare these extract systems further, we used label-free high-resolution mass spectrometry (HRMS) to identify relative differences in protein abundance.

### Proteomics comparison of the *E. coli* and *K. pneumoniae* CFE systems

**Fig. 2:**
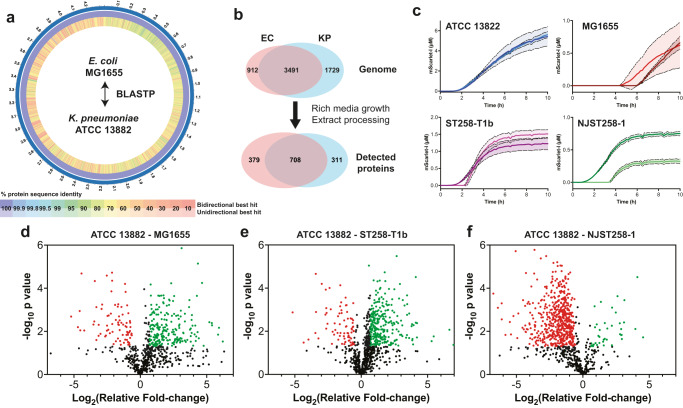
Comparison of *E. coli* and *K. pneumoniae* laboratory and clinical isolates. **a** Comparison of the *E. coli* MG1655 and *K. pneumoniae* reference genomes for protein homologues. Analysis was performed by the BV-BRC server^49^. **b** Genome predicted proteins for *E. coli* MG1655 (EC) and *K. pneumoniae* (KP) reference genomes (see “Methods”) and proteins detected by HRMS for MG1655 and ATCC 13882. **c** Real-time mScarlet-I synthesis from ATCC 13882 (blue and light blue line), MG1655 (red and dark red line), ST258-T1b (purple and pink line) and NJST258-1 (green and dark green line). Data for two independent extracts is shown, along with standard deviation (dotted lines and shaded area) for each extract representative of three repeat measurements. **d** Volcano plot of proteins showing either increased (green) or decreased (red) abundance changes for *K. pneumoniae* ATCC 13822 in comparison to *E. coli* MG1655. **e** Volcano plot comparison of *K. pneumoniae* ATCC 13822 and ST258-T1b cell extract proteins. **f** Volcano plot comparison of *K. pneumoniae* ATCC 13822 and NJST258-1 cell extract proteins. All proteomics data is an average of three independent samples. For full data, please refer to Supplementary File 2.

To provide a benchmark comparison, we selected the *E. coli* K-12 MG1655 laboratory model. The *E. coli* MG1655 genome is 4.6 Mb in size with a G+C content of 50.5% and encodes 4403 proteins (GenBank Accession: U0096; UniProt ID: UP000000625). *K. pneumoniae* ATCC 13822 (C37) has a 5.7 Mb genome with 56.5% G+C content and encodes 5241 proteins (GenBank Accession: GCA_017291375.1). Since the *K. pneumoniae* ATCC 13822 genome is not fully assembled, we used the multidrug-resistant HS11286 strain (Chromosome 5.3 Mb, 57.5% G+C content) as the closest reference^48^. The HS11286 chromosome (GenBank Accession: GCA_000240185.2; UniProt ID: UP000007841) is predicted to encode 5283 proteins and carries six plasmids that encode an additional 445 proteins in total, including several resistance proteins. We then compared the HS11286 and MG1655 UniProt reference datasets using a bidirectional BLASPT alignment via the Bacterial and Viral Bioinformatics Resource Center (BV-BRC) server^49^. This identified 3491 protein homologues shared between MG1655 and HS11286 (Fig. 2a, b), which ranged from 18.9 to 100% amino acid (aa) identity, with a mean identity of 76.85%. Sequence coverage ranged from 30 to 99.99% with a mean of 95.79% (Supplementary File 1); 912 and 1729 proteins are unique to *E. coli* MG1655 and *K. pneumoniae* HS11286, respectively (Fig. 2b). The remaining 508 proteins from *K. pneumoniae* proteome were duplicate matches to the *E. coli* database.

**Fig. 3:**
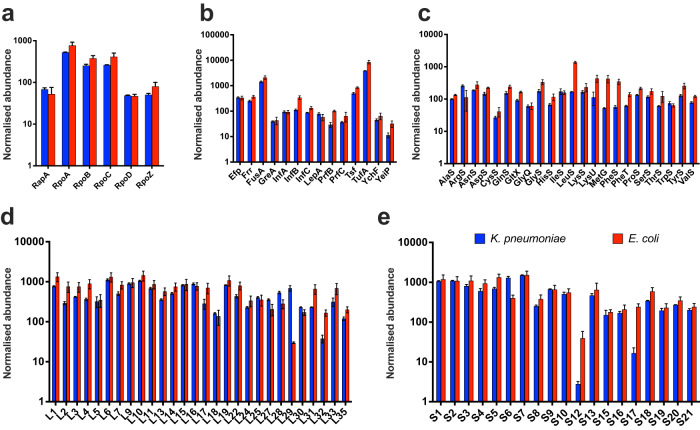
Normalised protein abundance of components from gene expression in *E. coli* MG1655 and *K. pneumoniae* ATCC 13882 cell extracts. **a** Transcription proteins. **b** Translation factors. **c** Aminoacyl-tRNA synthetases. **d** 50S ribosomal subunit. **e** 30S ribosomal subunit. Any protein that was not detected in one or more datasets was not compared. All data are included in the Supplementary File 2. *E. coli* MG1655 proteins are red. *K. pneumoniae* ATCC 13882 is blue. Please refer to “Methods” for information on data analysis. Error bars represent the standard deviation of three distinct extract samples.

We then compared the real-time CFE activity of the *E. coli* MG1655 and the *K. pneumoniae* CFE systems (Fig. 2c) under equivalent conditions using the mScarlet-I plasmid reporter (pSJM1174). An *E. coli* MG1655 CFE system was prepared as previously described^31^. In terms of relative protein synthesis activity, the *E. coli* MG1655 CFE system produced up to 1.3 μM of mScarlet-I after 10 h (Fig. 2c). The activity is modest because this K-12 strain lacks the genetic modifications that commercial B strains have for enhanced protein production, and the plasmid is not optimised for *E. coli* gene expression. In contrast, *K. pneumoniae* ATCC 13822 synthesised up to 6.7 μM mScarlet-I, and the ST258-T1b and NJST258-1 extracts displayed less activity, consistent with earlier observations. To study differences in the cell-free proteome, we digested the extracts with trypsin and detected proteins using label-free HRMS (See “Methods” for details). We digested three distinct cell extracts each for *E. coli* MG1655 and the *K. pneumoniae* ATCC 13882, ST258-T1b and NJST258-1 strains. Focusing on *E. coli* MG1655 and the *K. pneumoniae* ATCC 13882, 708 proteins were detected with homologues in both extracts, with a further 311 proteins unique to *K. pneumoniae* and 379 unique to *E. coli* (Fig. 2b and Supplementary File 2). Of the shared proteins, this represented 69.5% of the total proteins identified from *K. pneumoniae* ATCC 13882. In terms of the gene expression proteins, overall, these were very broadly similar in abundance (Fig. 3). Those that showed at least a six-fold increased abundance in *E. coli* MG1655 cell extracts included the S12 ribosomal protein, LeuS (leucine-tRNA ligase), MetG (methionine-tRNA ligase) and PheS (phenylalanine-tRNA ligase). Similarly, L29 ribosomal protein and ribosomal RNA 30S methyltransferase A (RsmA) were 23-fold and 26-fold higher in the *K. pneumoniae* ATCC 13882 cell extracts. Outside of core gene expression components, the top ten common proteins (out of 708) displaying a significant increase in either the *E. coli* MG1655 or *K. pneumoniae* ATCC 13882 cell extracts are listed in Table 1, including uncharacterised proteins.

**Table 1:** Selection of proteins with the highest fold-change in normalised protein abundance comparing *E. coli* MG1655 to *K. pneumoniae* ATCC 13882 cell extracts.

Accession	Name	Peptide count	Unique peptides	Confidence score	*p*-value	Relative fold change	Description
***E. coli***—**Increased abundance**							
A0A0H3GRP4	IscU	3	2	12.72	0.04588	203.80	Iron-sulfur cluster assembly scaffold protein IscU
A0A0H3GSZ2	FumB	9	1	63.80	0.02388	137.35	Fumarate hydratase class I_ anaerobic
A0A0H3GS19	CmoA	4	1	23.66	0.02954	74.04	Carboxy-S-adenosyl-L-methionine synthase
A0A0H3GRA2	PutA	5	3	29.44	0.00681	60.65	Bifunctional protein PutA
A0A0H3GWA1	Mdh	14	12	109.32	0.00523	42.26	Malate dehydrogenase
A0A0H3GLH6	SthA	4	3	29.17	0.00410	41.75	Soluble pyridine nucleotide transhydrogenase
A0A0H3GR04	RsuA	3	2	17.77	0.00376	36.71	Ribosomal small subunit pseudouridine synthase A
A0A0H3H3K6	KdsD	8	6	55.58	0.00006	25.12	Arabinose 5-phosphate isomerase KdsD
***K. pneumoniae***—**Increased abundance**							
A0A0H3GRI2	PtsG	14	10	97.22	0.00075	0.10	PTS system glucose-specific EIICB component
A0A0H3GWB3	MreB	10	10	84.00	0.00005	0.09	Cell shape-determining protein MreB
A0A0H3GXX3	YqjD	5	5	35.52	0.00346	0.09	Uncharacterised protein YqjD
A0A0H3GX70	YiaF	4	3	31.45	0.00483	0.09	Uncharacterised protein YiaF
A0A0H3GPV6	HemX	8	7	45.36	0.00909	0.05	Heme binding protein
A0A0H3GWL2	RpmC	6	6	51.35	0.00897	0.04	50S ribosomal protein L29
A0A0H3GJ37	RsmA	9	7	66.05	0.00641	0.04	Ribosomal RNA small subunit methyltransferase A
A0A0H3GTV2	Rnk	6	6	54.35	0.00205	0.03	Regulator of nucleoside diphosphate kinase

Finally, for unique proteins, we grouped these for the enrichment of certain classes using PANTHER^50^. The most enriched proteins unique to the *E. coli* MG1655 cell extract were from hexitol (GO:0006059), polyol (GO:0046174, GO0019751) and alditol (GO:00/9400) metabolic pathways. For *K. pneumoniae* ATCC 13882, a number of metabolic pathways were enriched, including from D-gluconate (GO:0046177) catabolism, as well as ribosome biogenesis (GO:0042254) and cell cycle (GO:007049). There were also 23/129 proteins from amino acid biosynthesis (GO:008652) only detected in the *K. pneumoniae* cell extract (Supplementary File 3). For the clinical strains these extracts did show several differences to the ATCC 13882 cell extract. Notably, the abundance of most proteins was lower in the NJST258-1 strain, although this is likely due to challenges with extract preparation from this clinical isolate (Supplementary File 4). Interestingly, the HRMS data identified a single β-lactamase (UniProt: A0A0H3GN17) for the ST258-T1b strain, three β-lactamases (UniProt: A0A0H3H184, A0A0H3H688, A0A0H3GN17) and an aminoglycoside 3’-phosphotransferase (UniProt: A0A0H3GZY9) for NJST258-1. While the CFE experiments do not cover resistance factors or antibiotic targets involved with the cell wall or cell membrane, the variable resistance to amikacin (an aminoglycoside) between extracts (Supplementary Fig. 14) suggests the A0A0H3GZY9 resistance protein was insufficient or alternatively, does not recognise amikacin as a substrate^51^. Overall, the proteomics data show that *E. coli* and *K. pneumoniae* cell extracts are similar in terms of protein components from gene expression and identify distinct differences between the laboratory and clinical extract strains.

### *K. pneumoniae* CFE provides a homologous assay to compare whole-cell antimicrobial activity

**Fig. 4:**
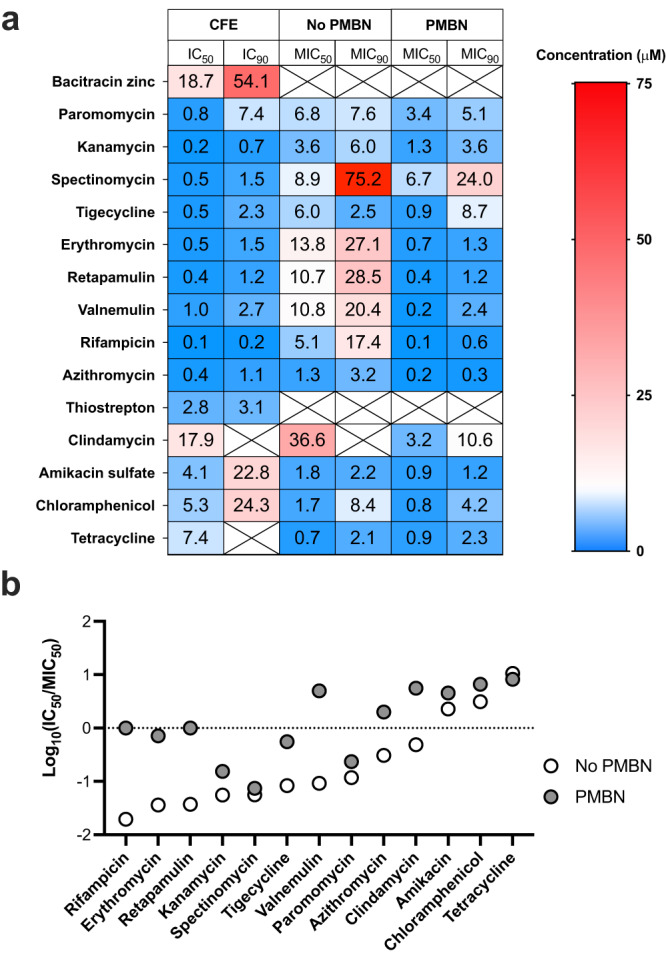
Antibiotic inhibition of *K. pneumoniae* cell-free and whole cells. **a** Summary of IC_50_ and MIC_50_ values. Whole-cell data was collected in the presence or absence of PMBN. Boxes with crosses represent undetermined inhibition values. Data is an average of three independent repeats. **b** Rank-ordered comparison of Log_10_(IC_50_/MIC_50_) ratios, where the MIC value is compared between the absence (white circle) and presence (grey filled circle) of PMBN. Data for thiostrepton, clindamycin and bacitracin zinc were removed because they were inactive in the whole-cell assays.

To provide a direct comparison of the native CFE system with cell-based growth inhibition in *K. pneumoniae*, we derived the minimal inhibitory concentrations for the respective assays. First, whole-cell assays were used to determine the minimum inhibitory concentration (MIC) value. MIC_90_ and MIC_50_ values were calculated as the concentration of antibiotic that caused 50% and 90% growth inhibition, respectively, for direct comparison with IC50 dose-response curves. For the CFE assay, the equivalent measure refers to the half maximal inhibitory concentration (IC) value that disrupts protein (mScarlet-I) synthesis as an estimate of IC_50_/IC_90_. We focused on the *K. pneumoniae* ATCC 13882 strain because of its strong CFE activity, and it is an established antimicrobial susceptibility testing standard and sensitive to most clinical antibiotic classes. Based on preliminary tests (Supplementary Fig. 15), we selected a total of 10 antibiotics, including azithromycin, retapamulin, valnemulin, thiostrepton, kanamycin, rifampicin, spectinomycin, paromomycin, erythromycin and tetracycline. We also included four antibiotics with weak activity in preliminary assays. These were tigecycline, amikacin, chloramphenicol, and clindamycin, which all target the ribosome. Additionally, bacitracin zinc was added as a non-specific CFE inhibitor since it targets cell wall biosynthesis. To explore limitations in antibiotic transport, we included polymyxin nonapeptide (PMBN) in whole-cell MIC assays to increase antibiotic import into the cells. We then performed bioactivity assays to quantify the IC_50_/IC_90_ and MIC_50_/MIC_90_ values (Fig. 4a) for these 15 antibiotics. For this, we used an 11-point serial dilution from 100 μM in 2% (v/v) DMSO. In addition, we also generated whole-cell bioassay data for the laboratory strains *E. coli* MG1655 and *K. pneumoniae* ATCC 13882, and the clinical isolates, *K. pneumoniae* M6, *K. pneumoniae* NCTC 13368, *A. baumannii* ATCC 17978, *E. coli* NCTC 12923, and *A. baumannii* AYE as a comparator for a range of Gram-negative laboratory and clinical isolates. This extended data is shown in Supplementary Table 2, which reveals a general trend in antibiotic resistance for all the clinical isolates in comparison to the *E. coli* MG1655 and the *K. pneumoniae* ATCC 13882 laboratory type strains.

Focusing on whole-cell and CFE data for *K. pneumoniae* (Fig. 4), 12 out of 15 compounds displayed a low IC_50_:MIC_50_ ratio (Fig. 4b) where the IC_50_ value was lower than MIC_50_ (Fig. 4 and Supplementary Fig. 16). In contrast, three compounds (chloramphenicol, amikacin sulphate and tetracycline) displayed a high IC_50_:MIC_50_ ratio (Supplementary Fig. 16a–c). At this point, we speculated that these values are linked to the antibiotic concentration inside/outside of the cell, while in the homologous CFE system this concentration is known. It is known that *E. coli* imports tetracycline and chloramphenicol faster than other common antibiotics^2^, with the latter being through an active transport system^52^. While the CFE system was sensitive to thiostrepton, bacitracin zinc and clindamycin showed limited activity, and none were effective in whole-cell assays.

Next, we repeated the whole-cell assay in the presence of polymyxin nonapeptide (PMBN), which increases the permeability of the cell membrane. Indeed, this sensitised the cells for thiostrepton (1.1 μM MIC_50_) and clindamycin (7.5 μM MIC_50_) but not bacitracin zinc (Supplementary Fig. 16d–f). For azithromycin, rifampicin, retapamulin, valnemulin, erythromycin, spectinomycin, kanamycin, paromomycin and tigecycline, the IC_50_ value was at least an order of magnitude lower than MIC_50_ (Supplementary Fig. 16g–o). For seven of these compounds, the MIC_50_ value decreased upon the addition of PMBN (Fig. 4). For five compounds within this set, the dose-response curve mirrors the CFE and PMBN data (Supplementary Fig. 16g–k). This provides evidence that for these classes of antibiotics, CFE provides a close bioactivity model for the membrane-permeability assay. Put together, the CFE and cell data highlight specific differences in antimicrobial activity for several antibiotic classes (Fig. 4), with the CFE approach providing a host-specific tool for exploring antimicrobial activity inside the cell.

### CFE profiling of laboratory-evolved AMR variants

Finally, we hypothesised that CFE could be used as a tool to study target mutations associated with AMR within a macromolecular biochemical environment that mimics some of the core processes inside a living cell without the presence of the membrane influx/efflux barriers. However, due to methodology limitations with the extracts of clinical strains (Supplementary Fig. 14), we took a bottom-up approach to engineer antibiotic resistance with the ATCC 13882 system. Here we created isogenic resistant strains to enable us to compare the effect of resistance-causing mutations in the same strain background. To do this, we applied adaptive laboratory evolution to generate stable mutants^53^. We serially passaged the ATCC 13882 strain against a selection of antibiotics that were characterised in the CFE and whole-cell assays (Supplementary Table 3). ATCC 13882 was serially passaged with increasing concentrations of antibiotics, starting at 0.25xMIC and doubling every 2 days until the bacteria were exposed to 4xMIC. Then, the strains were passaged 10x in the absence of antibiotics and the elevated MICs were retained in each case. We then analysed each strain by WGS analysis.

As expected, most antibiotic-resistant strains (e.g., tetracycline, kanamycin) accumulated multiple mutations, some in transport proteins, which were not suitable for further experiments (Supplementary Table 3). However, the rifampicin, chloramphenicol and valnemulin-resistant strains contained only one or two genomic mutations, which we prioritised for further study. Focusing on these strains for comparability to the ATCC 13882 strain, the MIC increased from 25 μM for valnemulin and 12.5 μM for rifampicin and chloramphenicol to >200 μM for all three antibiotics. These adapted strains are referred to as resistant to rifampicin (Rif^R^), valnemulin (Val^R^) and chloramphenicol (Chl^R^) derivatives of ATCC 13882. From WGS analysis, the Rif^R^ strain carries a single variant (H526L) in RNA polymerase beta subunit (*rpoB*), a mutation also observed in *Mycobacterium tuberculosis* Rif^R^ clinical isolates^54,55^. H526L is predicted to reduce the binding affinity of rifampicin to RpoB^54^. The Val^R^ strain had a single point mutation (AAC > TAC) at position 445 for *rplC*, which encodes the 50S ribosomal protein L3. Variants at the N149 position are associated with increased valnemulin and linezolid resistance in *E. coli*, with the L3 subunit located close to the peptidyl transferase centre (PTC) in the 50S large ribosomal subunit and the linezolid/valnemulin binding site^56,57^. Finally, the Chl^R^ strain carries a 24-bp repeat (duplication of amino acids 62–69) in an efflux pump regulator (OqxR) and an I264L variant encoded by a mutated (A790C) hypothetical gene [Locus_tag: WM93_12915, annotated as a histone-like deacetylase (HDAC), class II in related *K. pneumoniae* strain ATCC 35657].

**Fig. 5:**
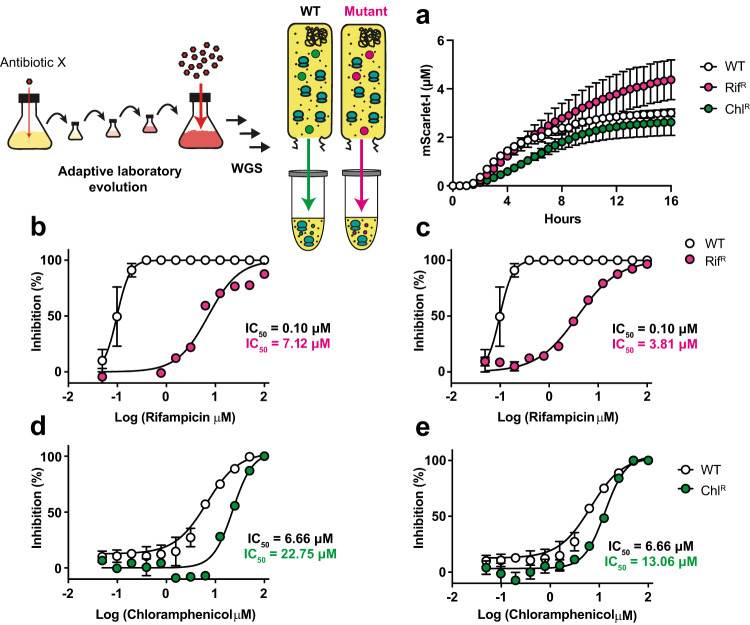
Adaptive laboratory evolution and characterisation of antibiotic resistance in *K. pneumoniae* CFE. **a** Real-time protein synthesis of mScarlet (μM) for the wild type (WT), Rif^R^ and Chl^R^ mutants. **b**, **c** IC_50_ data of two biological repeats for the Rif^R^ mutant plotted separately versus a representative dataset for the WT in response to rifampicin dosing. CFE data for WT is the mean and standard deviation of two independent experiments. **d**, **e** IC_50_ data of two independent extracts for the Chl^R^ mutant plotted separately versus a representative dataset for the WT in response to chloramphenicol dosing. CFE data is presented as a mean and standard deviation of three measurements from each extract.

To test the antibiotic-resistant mutant strains for CFE activity and for relative changes in protein synthesis in response to the corresponding antibiotic, cell extracts were generated (two biological repeats) and an IC_50_ was determined for each specific antibiotic and compared to the ATCC 13882 CFE system. Additionally, we also monitored mScarlet-I synthesis in real time in the absence of antibiotics (Fig. 5a). First, the Rif^R^ variant (RpoB H526L) displayed a 58-fold increase in IC_50_ (*p-*value = 0.0201) for rifampicin, compared to the parental strain (Fig. 5b, c). There were no apparent changes in protein synthesis rates between the ATCC 13882 and Rif^R^ CFE system. This was consistent across two biological repeats, suggesting that the H526L variant directly confers resistance to rifampicin, which agrees with previous studies^54,55^. For the Val^R^ CFE system, surprisingly, there was no apparent change in IC_50_ when valnemulin was added (Supplementary Fig. 17). While there was a slight delay in protein synthesis in comparison to the ATCC 13882 CFE system, the final yield of protein was similar (Supplementary Fig. 18). The unaltered IC_50_ value was unexpected, given that the RplC N149Y variant occurs close to the valnemulin and linezolid binding site, and that this was the only mutation identified from WGS analysis of this strain. Last, the Chl^R^ CFE system showed a significant increase in the IC_50_ value for chloramphenicol determined at 16.3 μM (*p*-value = 0.0154), in comparison to 5.3 μM measured for the ATCC 13882 strain (Fig. 5d, e). This might implicate that the histone-like deacetylase (HDAC) homologue (WM93_12915) somehow exerts a modest effect on chloramphenicol inhibition in the CFE system through an unknown mechanism, while the greater increase in the MIC value is likely due to the upregulation of efflux pumps through the mutation in the *oqxR* regulator.

## Discussion

AMR is a global pandemic projected to cause 10 million annual deaths by 2050, with an estimated total economic cost of $100 trillion^58^. Therefore, it is vital to find new ways to explore AMR, as well as develop novel counter strategies (e.g., non-standard antimicrobials, phage therapy, host-directed therapeutics) to fight resistance. Here we establish a *K. pneumoniae* CFE system, which provides a fast and safe model to study AMR genotypes from a representative model of a WHO priority pathogen. While a similar approach can be performed with in vitro approaches (e.g., purified ribosomes, enzyme complexes and mutants), target inhibition of an isolated protein has so far failed to deliver new antibiotic candidates to the clinic, despite extensive high-throughput screening studies^59^. A limitation of our study is that CFE bypasses resistance mechanisms and antibiotic targets associated with the cell envelope. However, our cell-free assay provides a host-specific intracellular model composed of proteins, metabolites and RNA species native to *K. pneumoniae*, as well as offering a safe biochemical model for this BSF-2 microbe. Our proteomics data also suggests that, while this native CFE system shares similarities with *E. coli* CFE, it differs in terms of the copy number of proteins involved in gene expression and unique proteins (many of unknown function) present under the growth conditions tested.

Typically, CFE systems used in antimicrobial characterisation have focused on laboratory models such as *E. coli* and *B. subtilis*^25,26^. Recent advances in cell-free synthetic biology suggest there is an opportunity to study almost any cell type that can be grown in the laboratory. This includes prokaryotes^22,30–32,34,35^ and eukaryotes^60–62^. After the initiation of our study, a *K. pneumoniae* CFE system was also recently released^63^, but alternatively, this work focused on the potential of this cell-free system for biotechnology and protein production. In contrast, we provide a *K. pneumoniae* CFE system for studying antimicrobials and intracellular AMR factors. Potentially this concept is generalisable to both current and emerging CFE model systems. By extending our studies to whole-cell MIC assays with a wide range of clinical antibiotics, we highlight the potential benefits of quantifying the on-target inhibitory effects of antibiotics in comparison to whole-cell measurements. Understanding antibiotic transport remains a significant challenge in AMR research. It is particularly difficult to quantify antibiotic influx/efflux for Gram-negative bacteria considering the cell envelope, which is a two-membrane layer, and the periplasmic space^2^. Our data show that 12 out of 15 antibiotics display a low IC_50_:MIC_50_ ratio between the CFE and whole MIC assays for *K. pneumoniae*. As an exception, chloramphenicol and tetracycline display a high IC_50_:MIC_50_ ratio. Chloramphenicol and tetracycline are known to have a high accumulation rate for *E. coli* compared to other common types of antibiotics^2^, which for chloramphenicol, is linked to active uptake mechanisms^52^. If the concentration of these two antibiotics is higher inside the cell than outside, this may be linked to the high IC_50_:MIC_50_ ratio for these two examples. Unfortunately, there are no equivalent studies on quantifying antibiotic transport for *K. pneumoniae* to validate this. However, the native CFE system enables the characterisation of antimicrobial activity (and comparison to whole-cell activity) to quantify target inhibition at a host-specific level.

Distinctly, we provide an alternative approach to study AMR from an intracellular perspective. While the cell envelope plays a major role in AMR, we provide a system to directly study how resistance mutations associated with intracellular components alter gene expression. A CFE system provides a core proteome and metabolome to catalyse transcription, translation, energy regeneration and amino acid metabolism. Our proteomics data also suggests there are potentially other essential processes, such as RNA modification, protein degradation, folding chaperones and uncharacterised proteins, and associated protein-protein interactions, that remain to be studied within the context of a cell-free extract. For now, we focused on the study of three laboratory-adapted *K. pneumoniae* strains with either single or double genomic mutations associated with AMR. While the antibiotics used are not clinically relevant for *K. pneumoniae* treatment, they were selected based on their targets (e.g., ribosomes, RNAP) and low MIC value observed in the whole-cell characterisation. From this proof-of-principal data, we show how individual mutations influence gene expression and AMR. We confirm previous findings from Rif^R^ *M. tuberculosis*^54,55^ that the RNA polymerase beta subunit H526L variant confers resistance to rifampicin. For the Chl^R^ strain, it is known that HDAC has been implicated in the establishment of persistence and antibiotic tolerance in *Burkholderia thailandensis*^64^, which is consistent with the CFE phenotype observed here. If CFE can be used to study this complex regulation of bacterial metabolism, then this might be a valuable tool for developing new adjunct therapies to prevent antibiotic tolerance. Since the HDAC is a hypothetical protein, the precise intracellular resistance mechanism remains unclear at this stage. In contrast, the >16-fold increase in MIC appears to be linked to the 24-bp repeat (duplication of amino acids 62–69) within the gene encoding the OqxR regulator, a protein already associated with significantly upregulated expression of the OqxAB efflux pump^65^. Finally, for a Val^R^ strain, there was no apparent change in IC_50_ value in response to valnemulin. This was unexpected since the mutation site, L3 N149Y, is located close to the PTC, close to the valnemulin/linezolid binding region. While this specific mechanism remains unknown at this stage, overall, our findings demonstrate that CFE provides a powerful approach to investigate the effects of specific mutations in known antibiotic targets and may clarify specific functions which might not be evident from either whole-cell or single-target studies. Since the system provides the transcription-translation steps and metabolic processes unique to *K. pneumoniae*, the effects of mutations associated with these processes can be monitored with the final reaction—i.e., protein synthesis. We highlight the RpoB H526L variant specifically since this provides a clear phenotype-genotype link in a clinically relevant context in *M. tuberculosis*. Our study used lab-evolved strains and selected for isogenic mutants. Ongoing work is required to widen the spectrum of AMR variants to validate the approach. The current data suggests CFE has the potential to study specific AMR variants directly interacting with antibiotic binding, which may be beneficial for host-specific SAR studies performed at BSL-1, which is especially useful if the core antibiotic scaffold is known to be active with whole cells.

Overall, our CFE model provides a robust evaluation of intracellular antimicrobial targets and genetic determinants associated with AMR in *K. pneumoniae*, a major Gram-negative bacterial pathogen. While the methodology requires preparation in a BSL-2 laboratory, the extracts are non-living and suitable for long-term storage and thus safe for shipping and study in a BSL-1 lab. This is beneficial since BSF-2 facilities are a barrier for some chemistry and natural product labs developing novel antimicrobials. This leads to an accessible research tool for AMR research, which together with recent advances in cell-free synthetic biology, offers a new opportunity to explore non-model bacteria associated with AMR and infectious diseases.

## Methods

### Molecular biology, bacterial strains and whole-cell antimicrobial assays

Routine molecular biology was performed as previously described^66^. *E. coli* MG1655 and DH10β (NEB) strains were used for molecular biology. *K. pneumoniae* strain ATCC 13882 was purchased from Deutsche Sammlung Von Mikroorganismen and Zellkulturen (DSMZ). All mutant strains of *K. pneumoniae* ATCC 13882 were generated at UKHSA (Supplementary Table 3) using laboratory-adapted evolution and sequenced. *K. pneumoniae* clinical strains ST258-T1b and NJST258-1 were previously described^67,68^. The strains were maintained and grown in standard Luria-Bertani, or specific media described within the supporting information. Clinical strains used for minimum inhibitory concentration assays (*K. pneumoniae* M6 and NCTC 13368, *A. baumannii* ATCC 17978 and AYE (BAA-1710), *E. coli* NCTC 12923) were acquired from ATCC or NCTC and maintained on Tryptic Soy Broth. All mutant strains of *K. pneumoniae* ATCC 13882 were generated at UKHSA (Supplementary Table 3) using laboratory-adapted evolution and sequenced.

### *K. pneumoniae* cell extract preparation

Cell extract preparation consisted of the following steps: cell growth and harvest, cell washes with S30A (14 mM Mg-glutamate, 60 mM K-glutamate, 50 mM Tris, pH 7.7) and S30B (14 mM Mg-glutamate, 60 mM K-glutamate, 5 mM Tris, pH 8.2) buffers, lysis by sonication, run-off reaction, and when specified, dialysis. The protocol for crude extract preparation was adapted and modified from previous studies^31^^,^^69^. Specifically, *K. pneumoniae* ATCC 13882 or clinical isolates were grown overnight on LB agar at 30 °C. A single colony was then inoculated into 5 mL of LB and grown at 30 °C, 200 rpm for 6–8 h (unless specified otherwise). Then, 50 mL of fresh sterile media was inoculated with 250 μL of pre-culture and incubated at 30 °C, 200 rpm for 14–16 h. The following day, the overnight culture was diluted 1:100 in 500 μL growth media in Ultra-Yield™ (Thomson, USA) baffled flasks and incubated at 30 °C, 220 rpm until the OD_600_ reached 2.5–3.0. Cells were transferred into pre-chilled 250 mL centrifuge bottles and centrifuged (10,000×*g*, 4 °C, 12 min). The supernatant was discarded. Then, cells were washed with 20 mL S30A buffer, recombined into a 30 mL centrifuge bottle and centrifuged (15,000×*g*, 4 °C, 12 min). The supernatant was carefully discarded, and the wash step was repeated. The cell pellet was resuspended, aliquoted into 2 mL polypropylene (PP) tubes and centrifuged (18,000×*g*, 4 °C, 12 min). The residual supernatant was discarded using a pipette tip. Using a pipette with the tip cut to increase bore size, the cell pellets were combined into a 50 mL PP tube for cell lysis by sonication. Cells were sonicated in an ice-water bath, in either a 50 mL PP tube or aliquoted into 1.5 mL PP tubes. Settings used were: 20 kHz frequency, 50% amplitude, 10 s pulse on time, 10 s pulse off time, 500 J energy input/mL of wet-cell pellet. The lysate was centrifuged (18,000×*g*, 4 °C, 10 min), and the supernatants were pooled and then aliquoted into 1.5 mL PP tubes. The lysates were incubated at 37 °C for 60 min (unless otherwise stated) and then clarified by centrifugation (18,000×*g*, 4 °C, 10 min). When required, clarified lysate was pooled and dialysed for 3 h at 4 °C in S30B buffer. The extract was subsequently clarified by centrifugation (18,000×*g*, 4 °C, 10 min), aliquoted into 200–500 μL fractions and stored at −80 °C. The total protein concentration of the crude extract was estimated using Bradford assay with a bovine serum albumin (BSA) standard. Using this method, the typical protein concentration of crude extracts from *K. pneumoniae* was 20–24 mg/mL.

### *K. pneumoniae* CFE reactions

The CFE reactions consisted of the following final concentrations (unless otherwise stated): 50 mM HEPES, 30 mM 3-PGA, 1.5 mM ATP, 1.5 mM GTP, 0.75 mM CTP, 0.75 mM UTP, 1 mM amino acids, 0–10 mM magnesium glutamate, 0–200 mM potassium glutamate, 3% (w/v) polyethylene glycol 8000, 0.2 mg/mL *E. coli* tRNA, 0.26 mM CoA, 0.33 mM NAD, 0.75 mM cAMP, 0.068 mM folinic acid, 1 mM spermidine and 10 nM plasmid DNA. All chemicals were prepared, as previously described^69^. For end-point measurement of protein synthesis, reactions were performed in 22 or 33 μL reactions in PP tubes as specified. Reactions were incubated at 28 °C, 250 rpm for 6 h or overnight (14–16 h). For time-course measurement, the reaction mixture was transferred as 10 μL technical replicates in a 384-well black, clear flat-bottom microplate. Fluorescence measurements (excitation filter 544 and emission filter 620 nm, 10 nm bandwidth) were recorded in a microplate reader every 10 min or after end-point incubation at 28 °C with 5 s of 250 rpm orbital shaking prior to measurement. Calibration standards of purified N-terminal His_6_-tagged mScarlet-I were prepared in the reaction mixture as described previously^45^. For *E. coli* MG1655 CFE, extracts were prepared as previously described^70^.

### CFE IC_50_ characterisation

Select compounds (see “Results” and Supplementary Information) were tested between a range of 0.04 μM to 100 μM via serial dilution in 1% (v/v) DMSO to determine half maximal inhibitory concentration (IC_50_) values. CFE reactions were performed as described in the primary screen in three technical replicates and using two different extracts. Data are fitted to either a symmetric (4-parameter) or asymmetric (5-parameter) logistic regression model and visualised using GraphPad Prism version 9.0. The data were normalised to percentage inhibition of mScarlet-I synthesis to provide a relative comparison to the MIC assays and to estimate IC_50_/IC_90_ values.

### Whole-cell assays with minimum inhibitory evaluation (MIC assay)

In this study, the MIC_50_ and MIC_90_ values are defined as the concentration of compound needed to cause 50% and 90% inhibition, respectively. A single colony was taken from a freshly grown agar plate of each strain and inoculated into 3 mL of sterile tryptic soy broth. The pre-cultures were incubated at 37 °C for 16–18 h with shaking at 200 rpm and then diluted to an OD_600_ of 0.01, equivalent to 1 × 10^6^ colony-forming units (CFU) per mL. This was further subcultured into a sterile clear flat-bottom 96-well plate to 5 × 10^5^ CFU per mL and dosed with one compound per plate. Each compound was serially diluted in 100 μL of TSB broth to which 100 μL of culture was added. Wells containing media and compound dilution were included as negative control while wells with bacterial strain only were used as positive control. The plates were incubated at 37 °C. During this incubation, bacterial growth was determined by reading absorbance at 600 nm on a CLARIOstar microplate reader (BMG, LabTech) with an attached plate-stacker enabling measurements of optical density at 600 nm (OD_600_) every 30 min for a total of 20 h. Background signal was eliminated by subtracting absorbance in blank wells. Data were normalised to the OD_600_ of positive control wells or wells with the lowest concentration of compound (whichever was higher) and expressed as percentage growth. MIC cut-off values were determined according to Clinical and Laboratory Standards Institute (CLSI) guidelines. Data is fitted to either a symmetric (4-parameter) or asymmetric (5-parameter) logistic regression model and visualised using GraphPad Prism version 9.0.

### Sample preparation and in-solution digestion for untargeted proteomics

Protein concentrations of samples to be analysed were determined by Bradford assay. To 2.5 μg/μL protein sample, urea and Tris-HCl (pH 8.2) were added to a final concentration of 8 M and 50 mM, respectively. 100 μg protein was transferred to a microcentrifuge tube and 0.5 M DTT was added to give a final concentration of 5 mM. The sample was incubated at 56 °C for 25 min. Freshly made 0.5 M iodoacetamide was then added to a final concentration of 14 mM and incubated at room temperature in the dark for 30 min. This was then diluted at a ratio of 1:5 with 25 mM Tris-HCl (pH 8.2). Then, 2 μg sequencing-grade trypsin was added, followed by 1 μL of 1% (v/v) ProteaseMAX™ surfactant per 100 μL. The sample was incubated at 37 °C for 4 h. An additional 2 μg trypsin was added and the samples were incubated overnight at 37 °C. After overnight digestion, trifluoroacetic acid (TFA) was added to a final concentration of 0.4% (v/v) to quench any remaining trypsin activity. The digested and acidified protein samples were cleaned up using Pierce™ C18 Spin Tips (Thermo Scientific™) according to the manufacturer’s instructions. All centrifugation steps were carried out at 1500×*g* for 1 min. The C18 tips were washed twice with 200 μL of 50% (v/v) acetonitrile (ACN) and centrifuged (1500×*g*, 1 min), followed by two equilibration steps with 200 μL of 5% (v/v) ACN and 0.5% (v/v) TFA. The samples were loaded in 0.4% (v/v) TFA and centrifuged. The flow-through was recovered and loaded again. The tips were washed three times with 200 μL of 5% (v/v) ACN and 0.5% (v/v) TFA. The bound peptides were eluted with 20 μL of 70% (v/v) ACN. Elution was repeated with an additional 20 μL of 70% (v/v) ACN. The samples were evaporated to dryness using a vacuum centrifuge and resuspended in 15 μL of 5% (v/v) ACN and 0.1% (v/v) formic acid. Peptide concentration was estimated using a Nanodrop™ spectrophotometer and diluted to 1 μg/μL in 5% (v/v) ACN and 0.1% (v/v) formic acid. The samples were stored at −80 °C until nanoLC-MS proteome analysis.

### Mass spectrometry analysis

Peptide samples, equivalent to 1 μg, were spiked with 50 fmol BSA for absolute quantification. Peptides were separated on an Acquity UPLC® High Strength Silica (HSS) T3 column (Waters) 75 μm i.d. × 15 cm (1.8 μm, 100 A) using an Acquity M-Class UPLC (Waters), elution was performed with a linear gradient from 3 to 40% B over 115 min [solvent A = 0.1% (v/v) formic acid, solvent B = 0.1% (v/v) formic acid, acetonitrile] and the eluate directed via a nanospray source to a Synapt G2-Si (Waters) with data collected in UDMSe mode.

### Database search and analysis

For genomic analysis, protein homologues and sequence identity were analysed using the Bacterial and Viral Bioinformatics Resource Center^49^ server (https://www.bv-brc.org/). Mass spectrometry data were imported into the software package Progenesis QI for Proteomics (Non-Linear Dynamics) and searched against a protein database using an MSe Search algorithm with a false discovery rate (FDR) set to 4%. Progenesis QI software (Waters) provided quality control information and quantification of peptides. The peptides were assigned using the reference proteome “*Escherichia coli* (strain K12)” (proteome ID UP000000625—accessed 02-08-2023) and “*Klebsiella pneumoniae* subsp. *pneumoniae* (strain HS11286)” (proteome ID UP000007841—accessed 03-08-2023) from UNIPROT, using an MSe Search algorithm (Ion Accounting/Apex3D). Automatic alignment was performed, and peak picking was done by assessing all runs with a minimum chromatographic peak width of 0.05 min and maximum allowable ion charge of 6. Following peak picking, MSE fragmentation data was used to automatically identify peptides by the Progenesis software, accounting for trypsin cleavage, carbamidomethyl modifications to cysteine residues and methionine oxidation. Maximum protein mass was set to 250 kDa with a maximum of one missed cleavage allowed. For peptide and protein assignments, a minimum of three fragments per peptide was required and a minimum of five fragments per protein. All assigned proteins contained at least one unique peptide. Absolute quantification using Hi-N was conducted with 50 fmol BSA calibrant (accession number P02769). To determine the enrichment of specific protein families, detected proteins were clustered using PANTHER (Protein Analysis Through Evolutionary Relationships, http://pantherdb.org)^50^. Graphics were imported into Adobe Illustrator for figure presentation.

### Laboratory-adapted evolution and WGS

The *K. pneumoniae* ATCC 13882 strain was serially passaged against a range of antibiotics selected from the CFE and whole-cell assays (Supplementary Table 3). *K. pneumoniae* ATCC 13882 was serially passaged with increasing concentrations of antibiotics, starting at 0.25xMIC and doubling every 2 days, until the bacteria were exposed to 4xMIC. Then, the strains were passaged 10x in the absence of antibiotic, the MICs were measured again and the elevated MICs were retained in each case.

### Whole-genome sequencing

Genomic DNA was purified using a Wizard genomic DNA purification kit (Promega). DNA was tagged and multiplexed with the Nextera XT DNA kit (Illumina). Whole-genome sequencing of *K. pneumoniae* isolates was performed by UKHSA-GSDU (UK Health Security Agency Genomic Services and Development Unit) on an Illumina (HiSeq 2500) with paired-end read lengths of 150 bp. A minimum of 150 Mb of Q30 quality data were obtained for each isolate. FastQ files were quality-trimmed using Trimmomatic. SPAdes 3.15.3 was used to produce draft chromosomal assemblies, and contigs of less than 1 kb were filtered out. FastQ reads from antibiotic-exposed isolates were subsequently mapped to the genome sequence of pre-exposed strain ATCC 13882 using BWA-MEM (version 0.7.17). Bam format files were generated using Samtools (version 1.6), VCF files were constructed using SNIPPY (Version 4.6.0) GATK2 Unified Genotyper (version 0.0.7) (50). They were further filtered using the following filtering criteria to identify high-confidence SNPs: mapping quality, _60; genotype quality, 40; variant ratio, _0.9; read depth, _10. All the above-described sequencing analyses were performed using Galaxy (www.useglaxy.org). BAM files were visualised in Integrative Genomics Viewer (IGV) version 2.12.2 (Broad Institute).

### Reporting summary

Further information on research design is available in the Nature Research Reporting Summary linked to this article.

## Supplementary information

Supplementary Information

Supplementary File S1

Supplementary File S2

Supplementary File S3

Reporting Summary

## Data Availability

Genomic sequencing data are available on NCBI with the following accession numbers: JAWRKO000000000 (Val^R^), JAWRKP000000000 (Tet^R^), JAWRKQ000000000 (Rif^R^), JAWRKR000000000 (Par^R^), JAWRKT000000000 (Kan^R^), JAWRKU000000000 (Chl^R^), JAWRKV000000000 (control). Proteomics data are available on PRIDE with the following accession code: PXD046861 and PXD046864. Other datasets generated during and/or analysed during the current study are available from the corresponding author upon reasonable request.
