# Supplementary material for: A cell-free strategy for host-specific profiling of intracellular antibiotic sensitivity and resistance
Source: NPJ Antimicrob Resist. 2023 Dec 18;1:16. doi: 10.1038/s44259-023-00018-z (PMC11721408; doi:10.1038/s44259-023-00018-z)
Supplement: Supplementary file 1 — Supplementary Information [file 44259_2023_18_MOESM1_ESM.pdf]

Supporting information

A cell-free strategy for host-specific profiling intracellular antibiotic sensitivity and resistance

Kameshwari Chengan[1,5], Charlotte Hind[2], Maria Stanley[1], Matthew E. Wand[2], Lakshmeesha Nagappa[1,5], Kevin Howland[1], Tanith Hanson[1], Rubén Martín-Escolano[1], Anastasios D. Tsaousis[1], José A. Bengoechea[3], J. Mark Sutton[2], Christopher M. Smales[1,4], Simon J. Moore[5]

[1]School of Biosciences, Division of Natural Sciences, University of Kent, CT7 2NJ

[2]Technology Development Group, Research and Evaluation Division, UK Health Security Agency, Salisbury, SP4 0JG, United Kingdom.

[3]Wellcome-Wolfson Institute for Experimental Medicine, Queen's University Belfast, Belfast BT9 7BL, UK

[4]School of Biological and Behavioural Sciences, Queen Mary University of London, London E1 4NS

Supplementary Information – Table of Contents

Supplementary Figure 1	Growth optimisation I
Supplementary Figure 2	Growth optimisation II
Supplementary Figure 3	Growth optimisation III
Supplementary Figure 4	Time course showing synthesis of the mScarlet-I protein in *K. pneumoniae* ATCC 13882 CFE from extracts harvested at different growth stages
Supplementary Figure 5	Effect of growth temperature on *K. pneumoniae* ATCC 13882 CFE activity
Supplementary Figure 6	Optimisation of sonication energy input for *K. pneumoniae* ATCC 13882 CFE activity
Supplementary Figure 7	Optimisation of incubation (“run-off reaction”) post cell-lysis for *K. pneumoniae* ATCC 13882 CFE activity
Supplementary Figure 8	Effect of dialysis on *K. pneumoniae* ATCC 13882 CFE activity
Supplementary Figure 9	Optimisation of DNA concentration for optimal *K. pneumoniae* ATCC 13882 CFE activity
Supplementary Figure 10	Comparing the effect of nucleotide triphosphates (NTPs) or monophosphates (NMPs) as primary energy source in *K. pneumoniae* ATCC 13882 CFE activity of extracts harvested at different growth stages
Supplementary Figure 11	Optimisation of Mg-glutamate and K-glutamate concentrations for the *K. pneumoniae* ATCC 13882 cell extract used for library screening experiments
Supplementary Figure 12	Relative activity (mScarlet-I fluorescence) of extracts before and after filter-sterilisation
Supplementary Figure 13	Recovery of viable colony forming units from *K. pneumoniae* ATCC 13882 cell-extracts
Supplementary Figure 14	Antibiotic-resistance profiling for *K. pneumoniae* NJST258-1
Supplementary Figure 15	Preliminary antimicrobial inhibition of *K. pneumoniae* ATCC 13882 CFE
Supplementary Figure 16	Antibiotic inhibition of *K. pneumoniae* cell-free and whole cells
Supplementary Figure 17	Dose-response curve of valnemulin inhibition of the ATCC 13882 (WT) and Val^R CFE systems
Supplementary Figure 18	Real-time protein synthesis of the ATCC 13882 (WT) and Val^R CFE systems
Supplementary Table 1	Plasmids
Supplementary Table 2	Antibiotic inhibition data for Gram-negative MIC testing
Supplementary Table 3	Whole genome sequence summary of ATCC 13882 antibiotic resistant strains
Supplementary Data 1	Proteomics data – MG1655 and ATCC 13882 extracts
Supplementary Data 2	Protein classification and enrichment
Supplementary Data 3	Proteomics data – ATCC 13882 and clinical isolate extracts

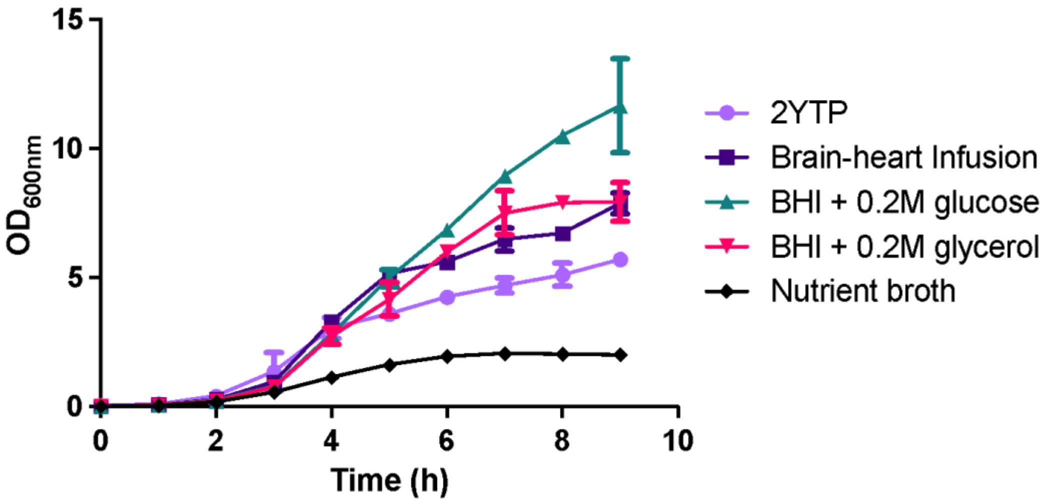

Supplementary Figure 1. Growth optimisation I. Growth curve of *K. pneumoniae* ATCC 13882 in various rich media. Cells were cultivated in 50 mL liquid cultures grown in 250 mL baffled flasks at 30°C, 200 rpm. Data is shown as mean ± standard error of mean (*n* = 2 biological repeats).

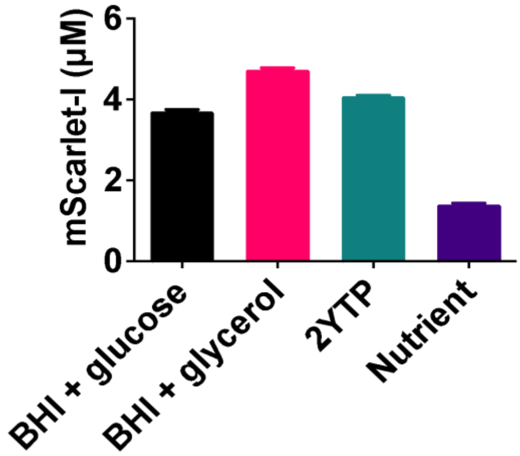

Supplementary Figure 2. Growth optimisation II. *K. pneumoniae* ATCC 13882 CFE activity from extracts generated from different growth media at an OD_{600} of 2.0. Cell-free reaction conditions: 8 mg/mL cell extract, 10 nM pTU1-A-SP44-mScarlet, standard energy solution, incubated at 30°C for 15 hours. Data is shown as mean ± standard error of three technical measurements.

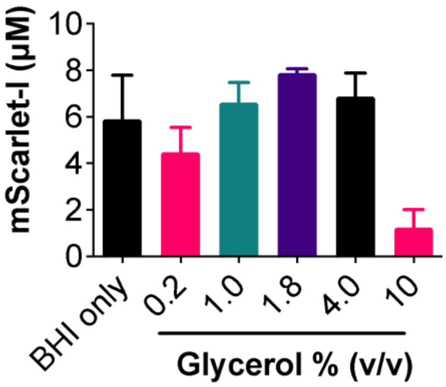

Supplementary Figure 3. Growth optimisation III. *K. pneumoniae* ATCC 13882 CFE activity from extracts grown in BHI supplemented with and without glycerol. Cells were cultivated in 50 mL BHI media with varying concentrations of glycerol at 30°C, 200 rpm, and harvested at an OD_{600} of 2.0. Cell-free reaction conditions: 8 mg/mL cell extract, 10 nM pTU1-A-SP44-mScarlet, standard energy solution and 30°C for 15 hours. Data was shown as mean ± standard error of mean (*n* = 2 biological repeats).

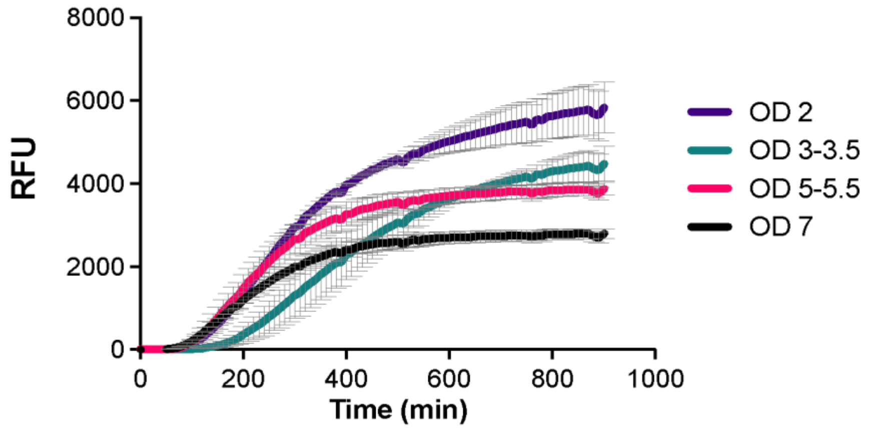

Supplementary Figure 4. Time course showing synthesis of the mScarlet-I protein in *K. pneumoniae* ATCC 13882 CFE from extracts harvested at different growth stages. Cells were grown in 50 mL BHI media with 1.8% glycerol (v/v/) at 30°C, 200 rpm and harvested at the OD_{600nm} indicated in the figure legend. Cell-free reaction conditions: 8 mg/mL cell extract, 10 nM pTU1-A-SP44-mScarlet-I, standard energy solution and 30°C for 15 hours, with fluorescence measurement every 10 min. Data is shown as mean ± standard error of mean (*n* = 2 biological repeats).

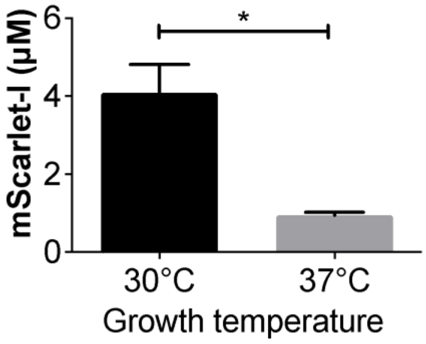

Supplementary Figure 5. Effect of growth temperature on *K. pneumoniae* ATCC 13882 CFE activity. Cells were cultivated from 50 mL cultures in BHI supplemented with 1.8% (v/v) glycerol, at 30 °C or 37 °C until an OD_{600} of 2.0-2.5 was reached. Data is shown as mean ± standard error (n = 2 biological repeats). *p<0.05 following a one-tailed unpaired *t*-test.

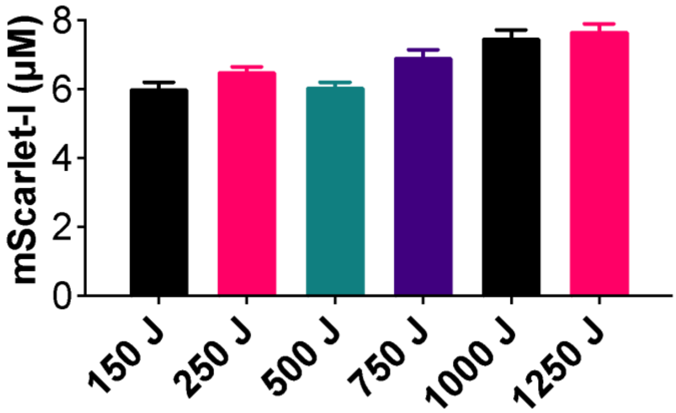

Supplementary Figure 6. Optimisation of sonication energy input for *K. pneumoniae* ATCC 13882 CFE activity. Cell-free reaction conditions: 8 mg/mL cell extract, 10 nM pTU1-A-SP44-mScarlet, standard energy solution and incubated at 30°C for 15 hours. Data is shown as mean ± standard deviation of three technical measurements.

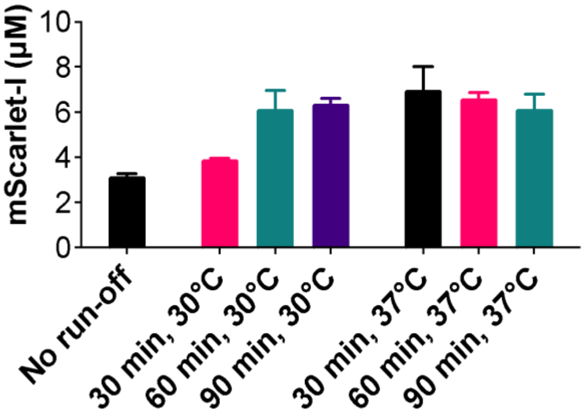

Supplementary Figure 7. Optimisation of incubation ("run-off reaction") post cell-lysis for *K. pneumoniae* ATCC 13882 CFE activity. Cell-free reaction conditions: 8 mg/mL cell extract, 10 nM pTU1-A-SP44-mScarlet, standard energy solution and 30°C for 15 hours. Data is shown as mean ± standard error of mean (n = 2 biological repeats).

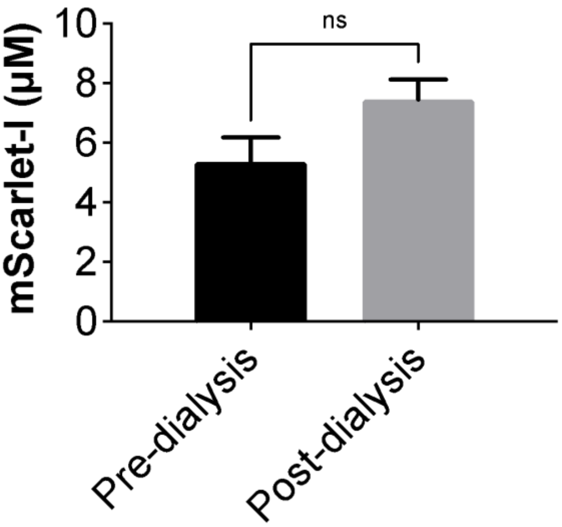

Supplementary Figure 8. Effect of dialysis on *K. pneumoniae* ATCC 13882 CFE activity. Cell-free reaction conditions: 8 mg/mL cell extract, 10 nM pTU1-A-SP44-mScarlet, standard energy solution and 30 °C for 15 hours. Extracts were dialysed against 1 L of S30B buffer for 3 h at 4 °C. Data is shown as mean ± standard error of mean for three biological repeats. P value > 0.05 following a two-tailed paired *t*-test (*n* = 3 biological repeats).

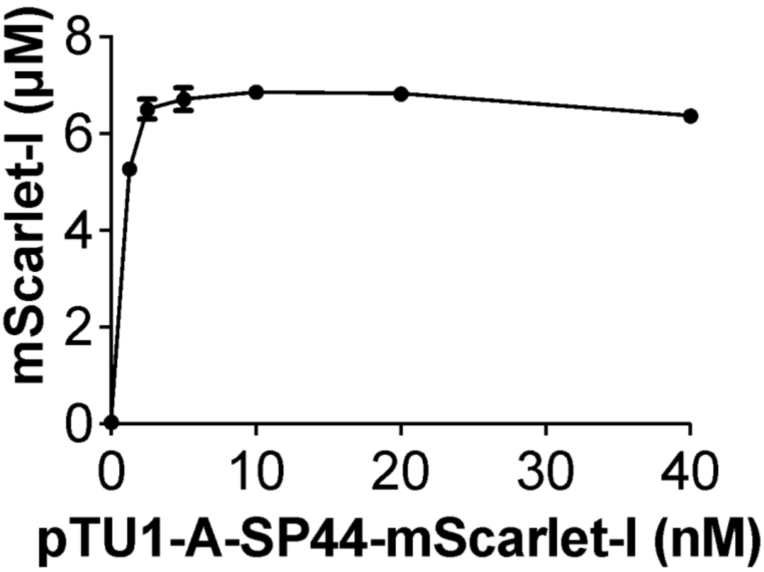

Supplementary Figure 9. Optimisation of DNA concentration for optimal *K. pneumoniae* ATCC 13882 CFE activity. Cell-free reaction conditions: 8 mg/mL cell extract, 0-40 nM pTU1-A-SP44-mScarlet, standard energy solution and 30°C for 15 hours. Data is shown as mean ± standard deviation of one representative dataset with three technical repeats (*n* = 2 biological repeats).

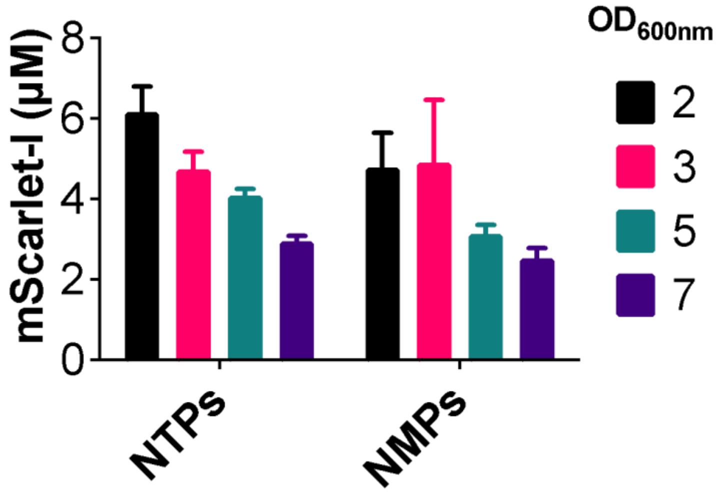

Supplementary Figure 10. Comparing the effect of nucleotide triphosphates (NTPs) or monophosphates (NMPs) as primary energy source in *K. pneumoniae* ATCC 13882 CFE activity of extracts harvested at different growth stages. Cell-free reaction conditions: 8 mg/mL cell extract, 10 nM pTU1-A-SP44-mScarlet, standard energy solution with either NTPs or NMPs and incubated at 30°C for 15 hours. Data is shown as mean ± standard error of mean (*n* = 2 biological repeats).

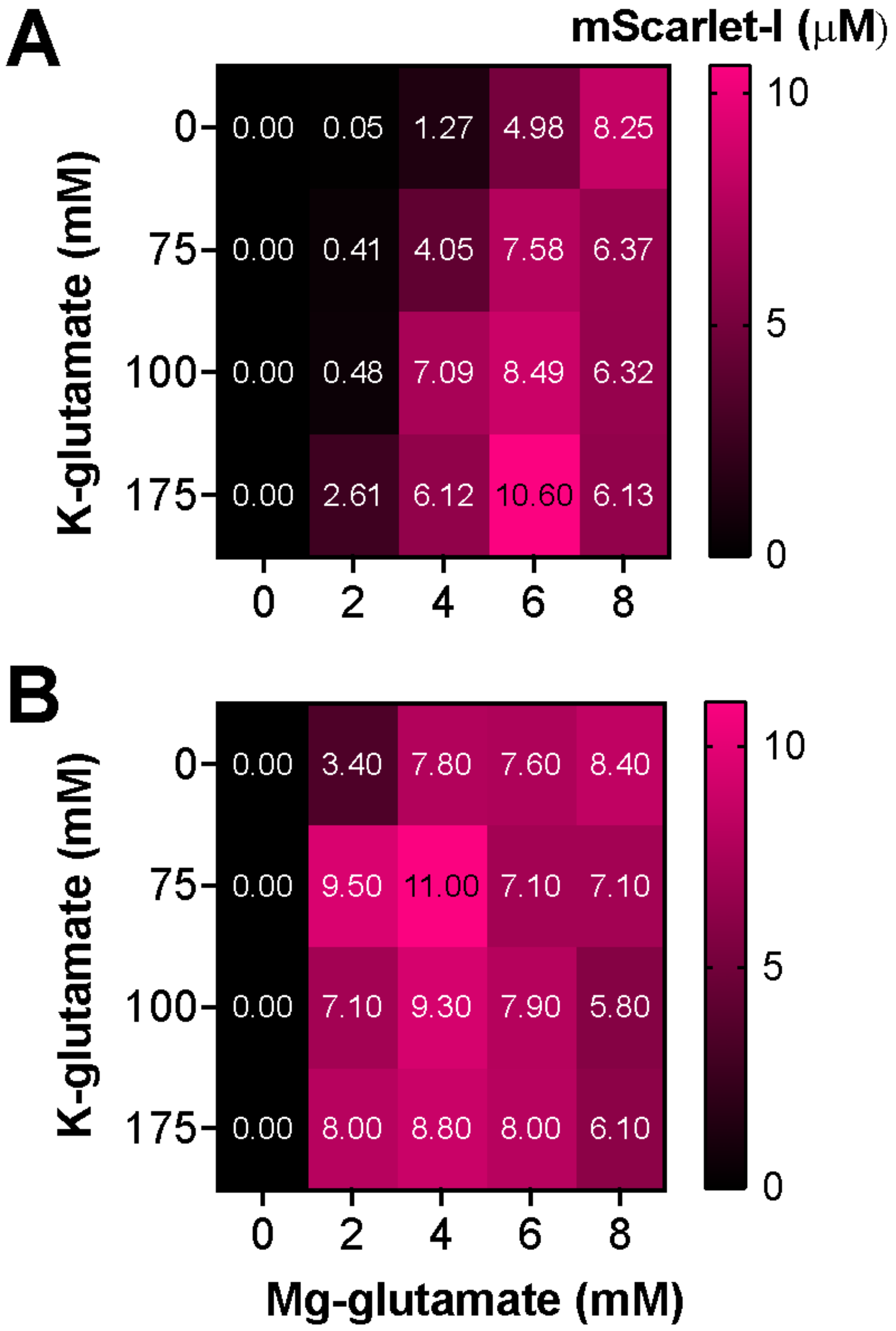

Supplementary Figure 11. Optimisation of Mg-glutamate and K-glutamate concentrations for the *K. pneumoniae* ATCC 13882 cell extract used for library screening experiments. Top (A) and bottom (B) panels show two independent batches of cell extract. Each time a new batch was prepared, this optimisation step was performed to ensure maximal activity and consistency between the experiments. Cell-free reaction conditions: 8 mg/mL cell extract, 10 nM pTU1-A-SP44-mScarlet, standard energy solution and 30°C for 15 hours. Data is shown as the mean of three technical repeats.

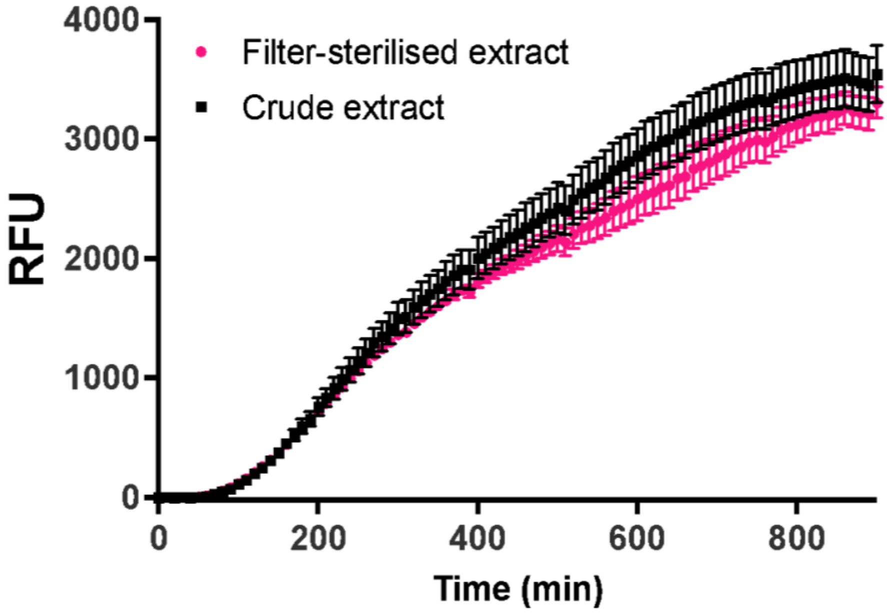

Supplementary Figure 12. Relative activity (mScarlet-I fluorescence) of extracts before and after filter-sterilisation. Reactions were prepared and measured using the standard methods outlined. Error bars represent standard deviation of three repeat measurements of a single extract.

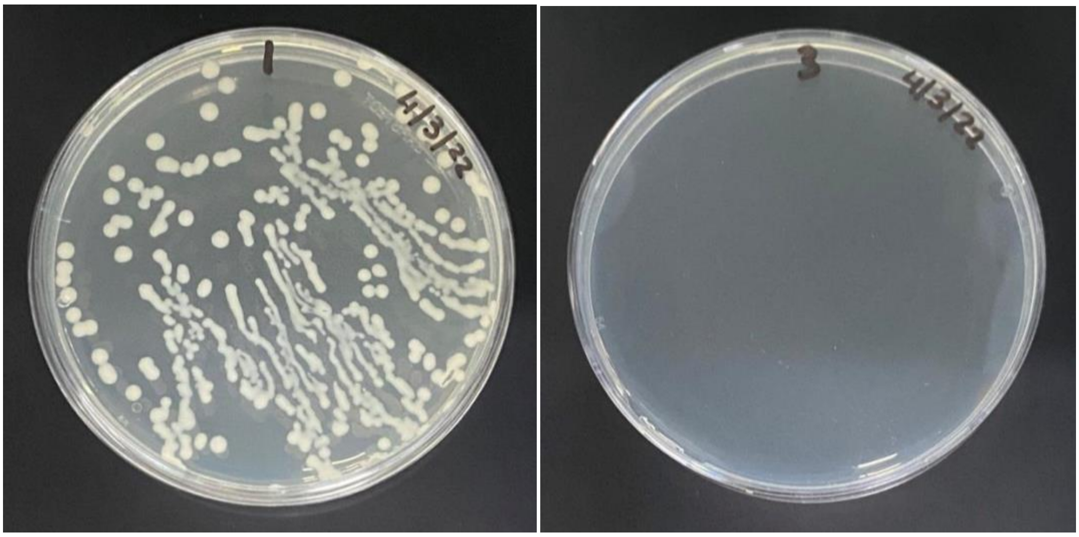

Supplementary Figure 13. Recovery of viable colony forming units from *K. pneumoniae* ATCC 13882 cell-extracts. 100 µL of 20 mg/mL cell-extract was spread onto a nutrient agar plate before (left panel) and after filtering (right panel) and incubated at 37ºC for 16 hours.

A Spectinomycin

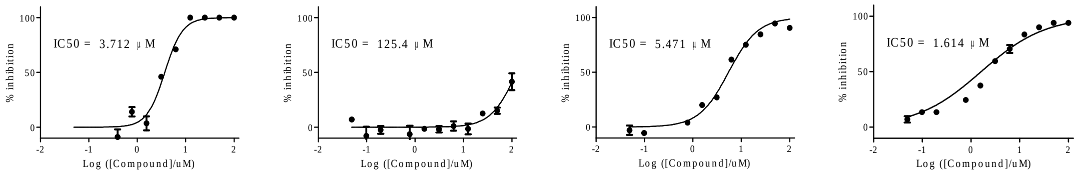

B Amikacin

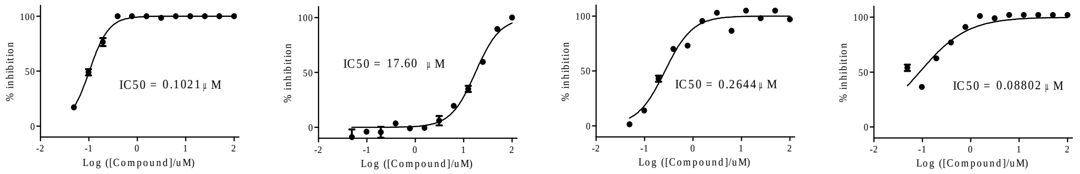

C Chloramphenicol

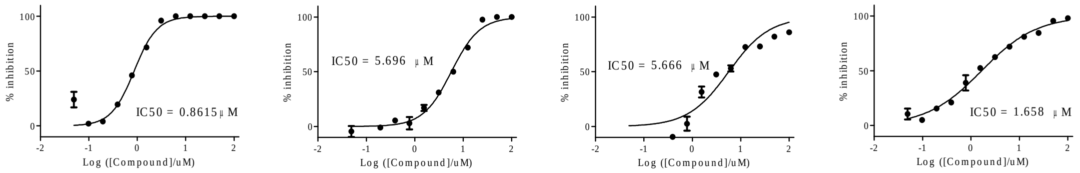

Supplementary Figure 14. Antibiotic-resistance profiling for *K. pneumoniae* NJST258-1. Predicted (based on RESfinder) resistance was tested for spectinomycin (**A**), amikacin (**B**) and chloramphenicol (**C**). Panels (left to right) represent four biological repeats of extracts grown under standard conditions in BHI medium. Note the extracts exhibit variable resistance, likely due to absence of positive selection during cell extract processing. Data is shown as mean and standard deviation of three technical replicates.

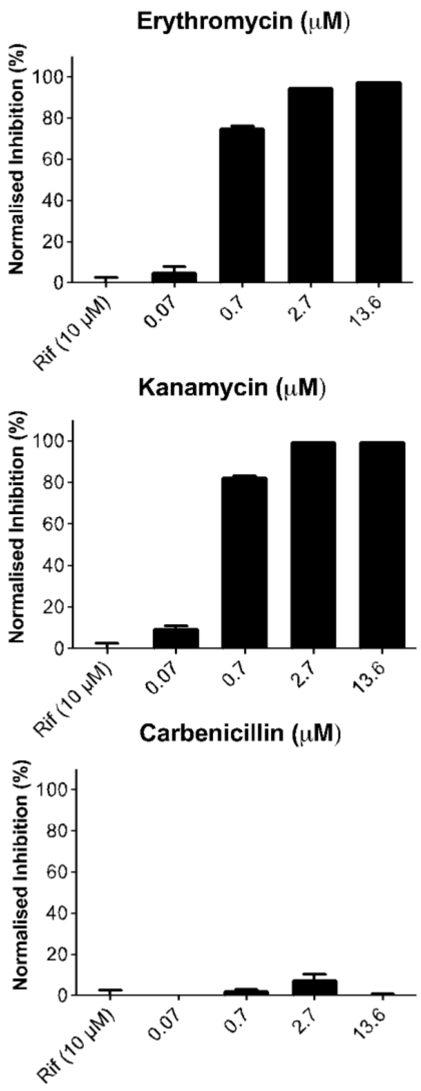

Supplementary Figure 15. Preliminary antimicrobial inhibition of *K. pneumoniae* ATCC 13882 CFE. Data presented as normalised activity (%) for three technical repeats.

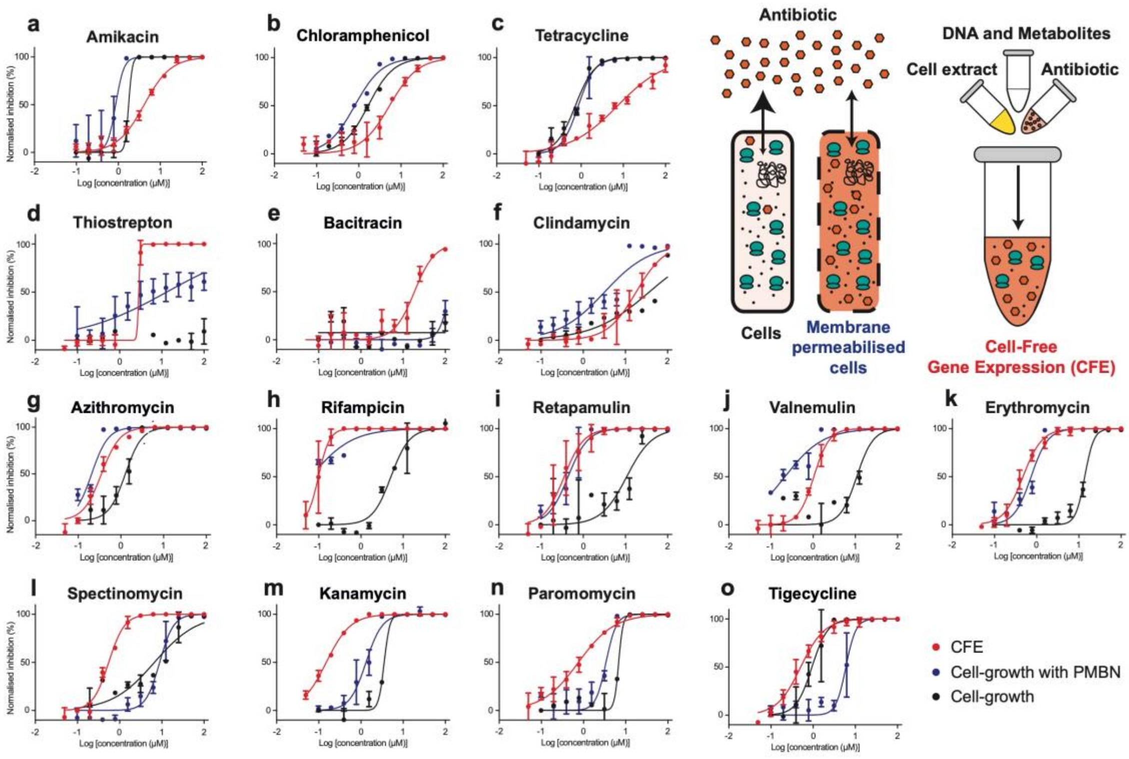

Supplementary Figure 16. Antibiotic inhibition of *K. pneumoniae* cell-free and whole cells. Antibiotics displaying a similar activity profile between the assays were grouped as follows: (**a-c**) Compounds that were more sensitive in whole cells; (**d-f**) Compounds with weak activity in cells; (**g-k**) Compounds with similar activity in cell-free and cells treated with PMBN; (**i-o**) Compounds with at least an order of magnitude higher sensitivity in cell-free. The data is normalised to provide a relative comparison of Log(concentration) versus normalised inhibition (%) between whole cell and cell-free assays. Data is presented as an average and error bars represent standard deviation. CFE data is the mean and standard deviation of two independent experiments, each with three technical repeats. Each whole cell assay data is mean and standard deviation from two independent experiments).

a

	WT	Val^R
IC50	1.039	0.8239

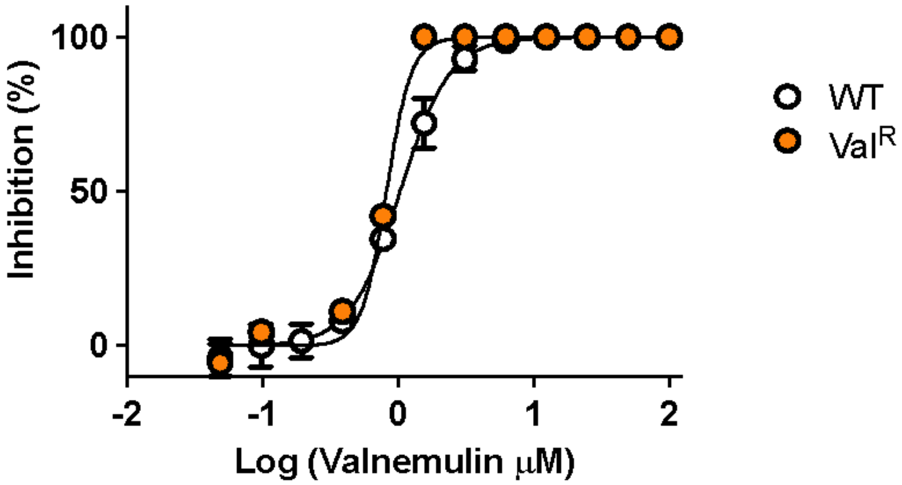

b

	WT	Val^R
IC50	1.039	0.6946

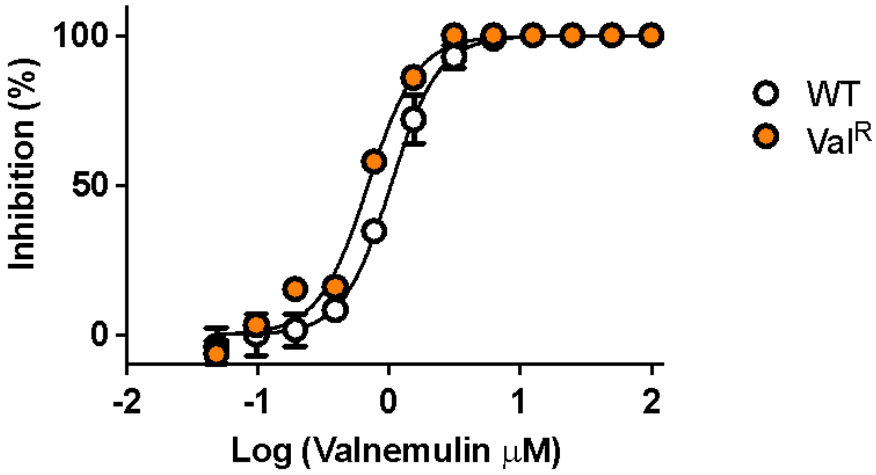

Supplementary Figure 17. Dose-response curve of valnemulin inhibition of the ATCC 13882 (WT) and Val^R CFE systems. Panels (a) and (b) represent two biological repeats, plotted separately, in comparison to a representative dataset for WT. Data for WT is shown as mean and standard error of mean of two independent experiments. See main methods for reaction setup and fluorescence settings.

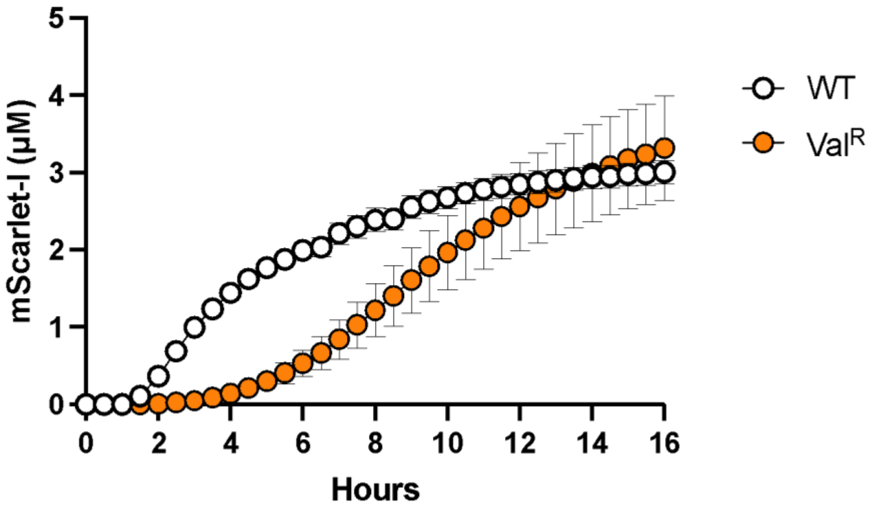

Supplementary Figure 18. Real-time protein synthesis of the ATCC 13882 (WT) and Val^{R} CFE systems. Error bars represent standard deviation of three repeat measurements of a single biological extract. See main methods for reaction setup and fluorescence settings.

Supplementary Table 1 – Plasmids

Plasmid name	Promoter	RBS	Gene	Terminator	AddGene No.
Pr-deGFP-MGapt	OR2-OR1-Pr	TTTGTTTAACTTTAAGAAGGAGA	deGFP	T7	#67734
pTU1-A-SP44-mScarlet-I	SP44	GTACTTTAACTTTAAGAAGGAGA	mScarlet-I	Bba_B0015	#163756

Supplementary Table 2

	K. pneumoniae DSM30102		*K. pneumoniae* M6		*K. pneumoniae* 13368		*E. coli* MG1655		*E. coli* 12923		*A. baumannii* 17978		*A. baumannii* AYE	
**PMBN added**	**No**	**Yes**	**No**	**Yes**	**No**	**Yes**	**No**	**Yes**	**No**	**Yes**	**No**	**Yes**	**No**	**Yes**
Tetracycline	2.0	2.2	3.4	7.8	54.6	38.0	5.4	3.8	4.5	3.6	2.7	8.4	>200.0	>200.0
Tigecycline	2.4	8.7	2.6	11.4	15.2	23.1	0.9	6.9	1.4	11.2	1.6	9.4	2.4	12.0
Amikacin sulphate	2.8	1.5	5.9	1.7	5.8	1.7	11.2	1.6	23.2	4.1	2.9	1.6	60.7	77.1
Azithromycin	3.0	0.4	6.0	0.5	27.6	2.0	3.1	0.1	5.8	0.2	0.1	0.1	15.2	4.0
Kanamycin	4.3	3.3	12.0	5.6	154.7	87.3	11.8	6.6	29.0	13.1	3.8	2.7	>200.0	>200.0
Choramphenicol	7.9	3.9	18.0	6.1	103.7	11.8	28.9	6.8	21.2	7.1	>200.0	>200.0	>200.0	>200.0
Paramomycin	11.4	5.8	11.5	11.0	>200.0	>200.0	24.6	11.3	74.6	17.9	10.5	4.6	>200.0	>200.0
Rifampicin	16.2	0.7	30.4	0.7	16.3	1.5	10.1	0.1	9.1	0.3	2.8	0.1	7.2	0.4
Valnemulin	21.2	2.0	80.9	8.3	138.0	16.5	28.9	0.7	108.0	0.9	13.5	0.1	24.8	0.8
Erythromycin	25.0	1.5	90.4	6.1	48.9	25.6	45.1	1.9	62.2	2.6	3.9	0.4	12.7	1.0
Retapamulin	31.0	1.2	113.1	6.2	>200.0	15.4	28.0	0.7	45.5	0.4	107.2	7.9	148.3	13.8
Spectinomycin	44.5	21.0	23.2	21.9	>200.0	47.4	41.4	59.7	155.6	101.0	48.9	41.8	>200.0	>200.0
Bacitracin zinc	>200.0	83.0	>200.0	>200.0	>200.0	32.0	>200.0	35.2	>200.0	39.1	40.6	40.6	38.1	52.6
Clindamycin	>200.0	12.4	76.8	18.1	112.2	56.7	>200.0	10.3	>200.0	7.0	55.9	49.1	55.6	8.0
Thiostrepton	>200.0	5.3	>200.0	6.9	>200.0	11.9	>200.0	34.3	>200.0	20.3	>200.0	0.4	>200.0	4.9

Antibiotic inhibition data was calculated using a Gompertz equation for MIC determination[1]. MIC (μM) values in excess of the concentration range tested, are resistant to the antibiotic (or >200 μM) tested.

Supplementary Table 3. Whole genome sequence summary of ATCC 13882 antibiotic resistant strains.

Adaptation	Gene	Mutation	Locus	Function	MIC (µM)	MIC after passage (µM)
Untreated control	*wcaJ*	Y69C	WM93_23710	undecaprenyl-phosphate glucose phosphotransferase	-	-
Rifampicin	*rpoB*	H526L	WM93_07445	DNA-directed RNA polymerase subunit beta	12.5	>200
Kanamycin	*cyoA*	W138STOP	WM93_12140	cytochrome ubiquinol oxidase subunit II	6.25	50
	fusA	A592V	WM93_04045	elongation factor G		
Tetracycline	*spoT*	10 bp del starting n2030	WM93_05500	(p)ppGpp synthetase	3.13	12.5
	oqxR	24 bp insertion after n201 (duplication of n177-201)	WM93_00285	transcriptional regulator		
Chloramphenicol	-	I264L	WM93_12915	acetoin utilization protein	12.5	>200
	oqxR	24 bp insertion after n201 (duplication of n177-201)	WM93_00285	transcriptional regulator		
Valnemulin	*rplC*	N149Y	WM93_04015	50S ribosomal protein L3	25	>200
Paromomycin	*sbmA*	Del n109	WM93_11755	microcin B17 transporter	12.5	>200
	fusA	D585G	WM93_04045	elongation factor G		
	ubiB	9 bp del starting n39	WM93_07275	ubiquinone biosynthesis regulatory protein kinase		

K. pneumoniae ATCC 13882 adaptation. All Locus tags based off *K. pneumoniae* strain ATCC 35657 (CP015134.1). An untreated control was also included in experiment and sequence analysis to verify for random genomic mutations.
